# Evaluation of Senegal supply chain intervention on contraceptive stockouts using routine stock data

**DOI:** 10.1371/journal.pone.0236659

**Published:** 2020-08-03

**Authors:** Catarina Krug, Francesca L. Cavallaro, Kerry L. M. Wong, Antonio Gasparrini, Adama Faye, Caroline A. Lynch

**Affiliations:** 1 Department of Infectious Disease Epidemiology, London School of Hygiene & Tropical Medicine, London, United Kingdom; 2 Institute of Child Health, University College London, London, United Kingdom; 3 Department of Public Health Environments and Society, London School of Hygiene & Tropical Medicine, London, United Kingdom; 4 Centre for Statistical Methodology, London School of Hygiene & Tropical Medicine, London, United Kingdom; 5 Centre on Climate Change and Planetary Health, London School of Hygiene & Tropical Medicine, London, United Kingdom; 6 Institut Santé et Développement, Université Cheikh Anta Diop, Dakar, Senegal; Helen Keller International, SIERRA LEONE

## Abstract

**Background:**

Until 2011, stockouts of family planning commodities were common in Senegalese public health facilities. Recognizing the importance of addressing this problem, the Government of Senegal implemented the Informed Push Model (IPM) supply system, which involves logisticians to collect facility-level stock turnover data once a month and provide contraceptive supplies accordingly. The aims of this paper were to evaluate the impact of IPM on contraceptive availability and on stockout duration.

**Methods and findings:**

To estimate the impact of the IPM on contraceptive availability, stock card data were obtained from health facilities selected through multistage sampling. A total number of 103 health facilities pertaining to 27 districts and nine regions across the country participated in this project. We compared the odds of contraceptive stockouts within the health facilities on the 23 months after the intervention with the 18 months before. The analysis was performed with a logistic model of the monthly time-series. The odds of stockout for any of the five contraceptive products decreased during the 23 months post-intervention compared to the 18 months pre-intervention (odds ratio, 95%CI: 0.34, 0.22–0.51).

To evaluate the impact of the IPM on duration of stockouts, a mixed negative binomial zero-truncated regression analysis was performed. The IPM was not effective in reducing the duration of contraceptive stockouts (incidence rate ratio, 95%CI: 0.81, 0.24–2.7), except for the two long-acting contraceptives (intrauterine devices and implants). Our model predicted a decrease in stockout median duration from 23 pre- to 4 days post-intervention for intrauterine devices; and from 19 to 14 days for implants.

**Conclusions:**

We conclude that the IPM has resulted in greater efficiency in contraceptive stock management, increasing the availability of contraceptive methods in health facilities in Senegal. The IPM also resulted in decreased duration of stockouts for intrauterine devices and implants, but not for any of the short-acting contraception (pills and injectables).

## Introduction

In 2017, approximately one-fifth (22%) of women of reproductive age reported not using contraception even though they wanted to delay their next birth or stop childbearing [1], a decrease in unmet need from 29% in 2011 [2]. Modern contraceptive prevalence rate (**mCPR**) among married women of reproductive age was 26% in 2017 [1], an increase from 12% in 2011 [2]. These successful indicators for unmet need and mCPR come after the Government of Senegal recognized the importance of addressing unmet need for family planning, committing to the FP2020 global partnership, and setting an ambitious target of achieving 27% mCPR nationally by 2015, more than two times the mCPR at the time of that announcement. One component of this strategy was to improve the contraceptive supply chain with the aim of reducing stockouts of contraceptive products [3, 4].

In 2011, interviews with women in two districts of the capital Dakar revealed that stockouts of family planning commodities were common in public health facilities, with 84% of interviewees having experienced a stockout of their method of choice during the previous year [3]. Limited availability of contraceptives at public health facilities is an important barrier to family planning use by women who wish to space or limit their childbearing [5]. In addition, the availability of a diverse range of contraceptive methods allows women to choose the method that best fulfils their needs and circumstances, and is shown to further increase mCPR [5, 6]. Addressing supply chain performance is therefore key to ensuring women are able to access contraceptives, and importantly, the contraceptives they want to use [7].

Up until 2012, Senegalese public health facilities relied on a pull-based supply chain system to manage their contraceptive stock. Within this system, the National Supply Pharmacy distributed health products to regional warehouses (Regional Supply Pharmacies), then the Managers of each health district stockroom would to travel to the regional stockroom to collect contraceptives. Subsequently, the stockroom manager (or *dépositaire*) of the health facility would collect and pay for supplies ordered at the district level [4].

As part of a broader strategy to increase contraceptive use, the Senegalese Ministry of Health assessed the feasibility of the Informed Push Model (**IPM**), which involved private operators acting as third-party logisticians to collect facility-level stock turnover data and provide contraceptive supplies accordingly [3, 4]. Daff et al. [3] evaluated the impact of the IPM pilot intervention on contraceptive availability in the region of Dakar. The authors observed a higher percentage of facilities with stockouts in the district of Pikine (n = 14) compared to the neighbouring district of Guediawaye (n = 19), with no IPM intervention [3]. Based on the successful outcome of the pilot [3], IPM was rolled out nationally to all public health facilities between late 2012 and early 2015 with the aim of eliminating contraceptive stockouts [4].

Several other countries have implemented informed push supply chain models, such as Zambia [8], Zimbabwe [9, 10], Mozambique [11] and Nigeria [12], but there have been limited rigorous evaluations of supply chain interventions on availability of health products. Some limitations of the studies in Senegal and other countries arise from small sub-national study areas [3, 12, 13], and short time periods that may not capture longer-term effects of interventions [3, 8–10, 13]. The impact of supply chain interventions on different dimensions of stock availability such as overall availability, and duration of stockouts, is lacking. To address these gaps in the literature, we aim to evaluate the impact of the IPM on contraceptive availability using routine stock data from up to two years after implementation from health facilities across nine of the 14 Senegalese regions. Secondarily, we aim to evaluate the impact of IPM on the duration of stockouts.

## Methods

### Study setting

Senegal has approximately 16 million inhabitants and an average population density of 68 inhabitants per square kilometre [14]. It is classified as a low income country with an estimated gross national income per capita of 1,410 USD [14]. The country is divided in five zones (North, South, East, West and Centre) which are further divided into 14 administrative regions, the region of Dakar being the most populated. The fertility rate is high in Senegal, with an average of five children per woman [1]. While prevalence of modern contraceptive use is increasing, it remains relatively low (26%) compared to other countries [1]. Contraceptive users obtain their methods primarily from public health facilities (86%), with a very low percentage obtaining methods from private health facilities or other sources [1]. Contraceptive cost depends on method and site, varying from 203 to 764 CFA Francs (0.3 to 1.3 USD) in public health facilities and from 1,050 to 3,645 CFA Francs (1.8 to 6.2 USD) in private health facilities. Condoms (male and female) are generally free, and implants and intrauterine devices (**IUD**) are the most expensive methods [15].

The public health system consists of national, regional and district levels. It follows a pyramidal structure with one health centre normally located in an urban area of the district, each of which oversees health posts in more rural settings [15]. The majority of women access contraceptive services in public sector health facilities [15]. All contraceptive products can be provided at all facility levels [1], conditional on the presence of trained staff (for example, for IUD and implant insertion).

### Intervention

IPM was implemented at regional level in Senegal, with private operators selected at that level to collect contraceptives from the regional warehouse and distribute them to all health facilities (excluding hospitals) within the region. As part of IPM, private operators were to visit each health facility in the region once monthly to perform an inventory and re-supply of contraceptive stock based on consumption in last three months. If health facilities experienced stockouts of any products during the month, they could contact private operators to arrange an additional delivery. In contrast to the pre-existing supply chain system which required up-front payment for all products ordered, under IPM the initial stock was provided by the private operators on credit and health facilities paid only for the contraceptive products sold to women. Private operators received extensive training and mentorship from the contracting organisation [3, 13, 16].

Contraceptives provided through IPM were: the combined pill, progesterone-only pill, progesterone-only injectable, implant, IUD, male and female condoms, and fertility beads. The emergency contraceptive pill was also included during the course of the IPM intervention. These contraceptives were removed from the pre-existing supply chain, which continued to deliver all other medicines and medical supplies. IPM was rolled out nationally in a staggered manner to all public health facilities between late 2012 and early 2015. Implementation began in the most densely population regions of the West of the country, and subsequently reached the central, northern and southern regions [3, 4, 17, 18].

### Data sources and measurement

To estimate the impact of the IPM on product availability over time, stock card data were obtained from Senegalese health facilities in two data collection phases, one in 2015 and another in 2016. Stock cards are used by the stockroom manager in health facilities to record entry and exit per medical product in each health facility. Stock inventories, undertaken by district supervisors or by private operators as part of IPM were also recorded on the stock cards. During initial visits to health facilities, stock cards were identified as the most commonly used method to record medical commodities.

Selection of health facilities occurred through three-stage sampling. Firstly, one region was randomly selected among each of the four zones of Senegal–Centre (Diourbel, Fatick, Kaffrine, Kaolack), North (Saint Louis, Louga, Matam), South (Ziguinchor, Sedhiou, Kolda), and West (Dakar, Thies). A sampling frame, in which all districts in a given region were listed, was used to randomly select three health districts per region. For each district chosen, the health centre for the district was automatically included and three health posts were randomly chosen from the list of all operating health posts in the district. Hospitals were not included in the definition of our source population as they were not included in the IPM intervention.

The regions of Thies, Diourbel and Ziguinchor were visited in the first phase, while Dakar, Matam, Fatick, Kaffrine, Sedhiou and Kedougou were visited in the second phase. On average (range), 3.8 (2–6) facilities were selected per district, and 3.0 (2–4) districts were selected per region.

Data collection was undertaken by ten teams, each comprising of one supervisor and two surveyors previously selected and trained by the study group. The team scanned all product stock cards available from 24 months pre- to 24 months post-intervention. Preliminary analysis showed that data availability between 18 and 24 months prior to the intervention was severely limited, thus it was decided to truncate data to 18 months pre-intervention in the analysis. Stock card data was obtained for five contraceptive products in Senegal (combined pill, progesterone-only pill, progesterone-only injectable, implant and IUD). Although stock card data was available for all nine contraceptive products, a pilot study showed these were often unavailable for the remaining four contraceptives (male and female condoms, and fertility beads and emergency contraceptive), and it was therefore decided to scan only the four most commonly used contraceptives, as well as IUDs given the MoH focus on increasing long-acting reversible contraception use. The most commonly used methods among women of reproductive age in union are injectables, pills and implants [1, 2]. We also collected stock card data on potential comparison products (amoxicillin in syrup and artemisinin-based combination therapy for babies, **ACT**) delivered by the pre-existing supply chain and not exposed to the IPM intervention. These comparison products were therefore considered good proxies for trends for contraceptive commodities if the IPM had not been implemented. The scanned stock information was then inputted in Excel files by *ProcessWorks Company* (Bulgaria) after training and with extensive data quality checks by one author (FLC). Further information on how facilities were selected and how data were collected can be found in S1 File.

Ethical approval for this study was obtained from the ethics committee of the *Conseil National de Recherche en Santé* (CNRS) in Senegal (n° 107/MSAS/DPRS/CNERS), and from the London School of Hygiene & Tropical Medicine (Ethics ref: 9925).

### Statistical analysis

To address the first objective, we used an interrupted time series analysis with ‘any monthly stockout’ as the outcome and facility-month as the unit of statistical analysis; the second objective was addressed using a negative binomial regression model of duration of product stockout pre- versus post-intervention, with stockout-event per product within facility as the unit of statistical analysis.

A stockout event was defined as a product being unavailable (i.e. existing stock = 0) for one or more days in a facility. We made some assumptions about stock card data; for example, we assumed that when there was a time lag between two stock records (i.e. purchases, sales, or inventory), the stock available within the time lag was equal to the last recorded stock. For our first analysis, stockout event relates to any of the five contraceptives missing in a specific month, in the second analysis, stockout event relates to a specific product, during all months before or all months after the IPM.

For both analyses, facility, district and region were considered random intercepts to account for the higher correlation among repeated observations from the same facility, and observations from the same district or region. Zone was included in both models as a fixed effect to control for clustering of observations by zone. Data management and descriptive statistics were performed using STATA statistical software, version 14 (StataCorp). MLwiN version 2.33 [19] was used to conduct all the multilevel models adjusted for the design weights. Weights were calculated as shown in S2 File, and were indicated by the user for each level. MLwiN was instructed to also standardize or scale the weights, so that the new weights sum to the effective cluster size, as detailed by the Centre for Multilevel Modelling [19]. Standardized weights are recommended in multilevel models for less biased estimates compared to ‘raw’ weights [20, 21]. Consequently, all results are presented for models adjusted for standardized weights. The variance proportion was calculated as described by Dohoo et al. [22]. S3 File gives detail on study power.

### Stockout monthly occurrence

The primary outcome was any stockout within the month of any of the five contraceptives–combined pill, progesterone-only pill, progesterone-only injectable, implant and IUD. We created a binary outcome for ‘any monthly stockout’ taking the value ‘0’ if all five contraceptives were always present during that month (no stockouts) and ‘1’ if at least one stockout was recorded for at least one of the products. For example, if in a health facility, implants were out of stock during two days in a month, then for that specific month, in that facility, ‘any monthly stockout’ would be coded as ‘1’. The variable for monthly stockout for ACT and amoxicillin, was defined in a similar way, creating a stockout variable for each separately. The analyses were restricted to the 18 months pre- and 23 months post-intervention.

We conducted an interrupted time series analysis to assess the impact of the IPM on stockout events for the different products (contraceptives and comparison products), using generalized mixed models [23]. The predictors were time (from -18 to 23 months relative to the intervention), intervention (0 before and 1 after the intervention) and time*intervention (0 before and from 1 to 23 months after the intervention). The general model was the following:

$$Y_{ijkl}∼bin\left[P\left(Y_{ijkl}\right)\right]$$

$$Logit\;[P(Y_{ijkl})]=\beta_{0jkl}+\beta_{1}Time_{ijkl}+\beta_{2}Intervention_{ijkl}+\beta_{3}Time*Intervention_{ijkl}+\beta_{4}Zone+f_{l}+v_{kl}+u_{jkl}$$

where P(Y_ijkl_) is the probability of stockout for the i^th^ month from the j^th^ facility from the k^th^ district from the l^th^ region; β_0_ is the outcome at time 0; β_1_ reflects the overall effect of time (measured in elapsed months), β_2_ reflects the effect of the IPM on the odds of stockout, β_3_ reflects any change in time trend effect caused by the IPM; β_4_ is the zone fixed effect included to account for clustering of observations by zone; and f_l_, v_kl_ and u_jkl_ are the region, district and facility error terms, respectively (random parts of the model).

To evaluate the assumption of normality between time and the log odds, residuals were visually examined for each model using quantile–quantile plot. The assumption of homoscedasticity was assessed visually using plot of the residuals against predicted values.

### Product stockout duration

Stockout duration was defined as the number of consecutive days a product was stocked out. The start of a stockout event was defined as the day on which a product stock changing from positive to null (i.e. there was a sale of that product at the health facility), and the end was defined as the day with a recorded change to positive (i.e. there was a stock refill at the facility). For example, if in a health facility injectables were out of stock for 40 consecutive days (null), and after being re-stocked (positive) they experienced another stockout of 20 days (null), we would consider that facility had two stockout events for injectables: one of 40 days and another of 20 days duration. We restricted the analyses to the 18 months pre- and 18 months post-intervention due to the nature of the outcome. The stockout event could take any duration between 1 day and 18 months (i.e. the maximum follow-up time before IPM implementation and afterwards).

To evaluate the impact of the IPM intervention on stockout duration, since zero counts were not possible (i.e. zero days in stockout), a zero-truncated model was fitted to the data by subtracting one from the outcome and modelling the revised outcome [22]. The main predictor was intervention (0 before and 1 after the intervention) and an interaction term intervention*product was forced into the model to investigate the effect of the intervention for each contraceptive product. The model used for estimates of stockout duration was:

$$D_{ijkl}∼NegBinom\left(\lambda_{ijkl}\right)$$

$$ln(\lambda_{ijkl})=\beta_{0jkl}+\beta_{1}Intervention_{ijkl}+\beta_{2}Product_{ijkl}+\beta_{3}Intervention_{ijkl}*Product_{ijkl}+\beta_{4}Zone+f_{l}+v_{kl}+u_{jkl}$$

where D_ijkl_ is the number of days in stockout minus one and λ_ijkl_ is the expected number of days in stockout for a i^th^ event from a j^th^ facility, from the k^th^ district, from the l^th^ region; β_0_ is the intercept; β_1_ is the log incidence rate ratio (**IRR**) between pre and post-intervention; β_2_ is the effect of product; β_3_ is the intervention*product interaction; β_4_ is the zone fixed effect included to account for clustering of observations by zone; and f_l_, v_kl_ and u_jkl_ are the region, district, and facility error terms, respectively (random parts of the model). Parameter estimates from mixed eect generalized linear models represent the average of all the subject-specific estimates from model (average of all the health facilities and contraceptive products included in the study). Model predictions for stockout duration for IUD and implant were adjusted for standardized weights and derived using the simulation-based procedures of the MLwiN customised predictions facility.

## Results

**Fig 1:**
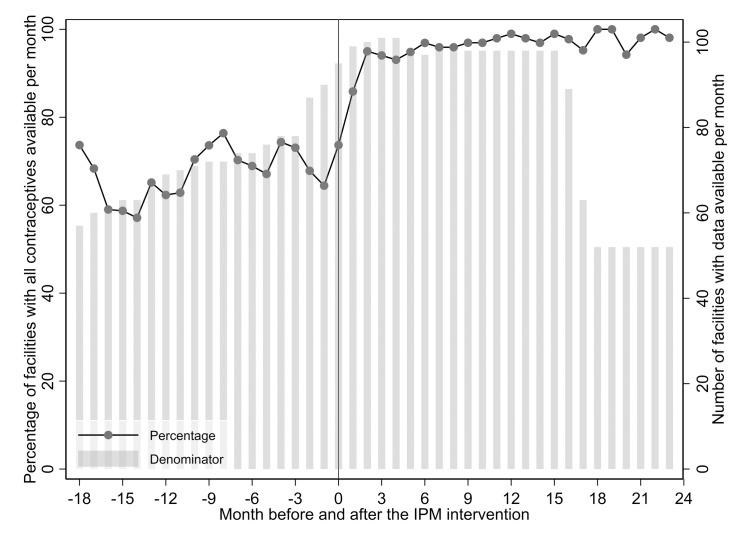
Health facilities with all contraceptives available during the months prior and subsequent to the intervention. The full line represents the percentage of facilities with all five contraceptives available per month, non-adjusted for sampling weights. The grey bars represent the denominator used to calculate the percentage at each month, which is the number of health facilities with data available, over a total of 103. Contraceptive products are combined pill, progesterone-only pill, injectable, implant and intrauterine device; IPM: Informed Push Model intervention; Intervention occurred at time 0.

In total, 103 health facilities (24 health centres and 79 health posts) in 27 health districts and nine regions of Senegal were included in this study. During the months before the intervention, 60 to 80% of facilities had all five contraceptives available in any given month, while after the intervention, that proportion increased to almost 100% (Fig 1).

### Stockout monthly occurrence

#### All five contraceptive products

Of 103 facilities, 95 had stock card data on contraceptives previous to the IPM. Before the intervention, the median number of months with available data for all five contraceptives was around 19 per facility across the 95 health facilities in the country. After the intervention, the median number of months with available data varied (within district) from 15 to 23 across the 103 health facilities. The number of facilities with data available per month dropped after 18 months because some facilities had their data collection 18 months after the IPM was implemented (Fig 1).

During the study period, there were 723 facility-months with at least one stockout (from a total of 3,317 facility-months with records), of which 225 (31%) occurred post-intervention. The regression analysis confirmed a reduction in ‘any monthly stockout’ before and after the IPM intervention. The odds of stockout decreased immediately after the intervention (odds ratio, **OR**, 95%CI: 0.34, 0.22–0.51), as shown by significance of β_2_. In other words, by calculating the reciprocal of the OR, we can say that there was a strong evidence of an immediate effect of the IPM in the odds of availability of the five contraceptives by 3 (OR = 1/0.34 or OR = e^1.09^). The odds of stockout remained consistent over time after the IPM (i.e. the post-intervention slope, β_3_, was not different from zero). Similarly, the increase in availability was not affected by any factors occurring before the intervention, as shown by the pre-intervention slope (β_1_; Table 1). Health facility characteristics (variance proportion = 100%) explained all the observed variation in the odds of ‘any monthly stockout’. District- or regional-level factors did not contribute to variation in ‘any monthly stockout’ (Table 1).

**Table 1:** The effect of the Informed Push Model intervention on odds of any monthly stockout for contraceptive and comparison products, in Senegalese health facilities.

Parameter	β^a^	SE^a^	*p*-value^a^	OR_stand_ (95%CI)^a^			OR_raw_ (95%CI)		
**Main outcome** (n = 3,317): Any of the five contraceptives^b^									
β_0_	-0.60	0.33							
β_1_Time	-0.01	0.02	0.617	0.99	(0.96,	1.0)	0.99	(0.95,	1.0)
β_2_Intervention	-1.09	0.21	<0.001	0.34	(0.22,	0.51)	0.34	(0.24,	0.48)
β_3_Time*Intervention	-0.03	0.02	0.285	0.98	(0.93,	1.0)	0.98	(0.93,	1.0)
Region-level variance	0.00	0.00							
District-level variance	0.00	0.00							
Facility-level variance	4.02	0.99							
**Comparison product 1** (n = 1,249): Amoxicillin syrup									
β_0_	-1.10	0.33							
β_1_Time	0.04	0.03	0.116	1.0	(0.99,	1.1)	1.0	(0.96,	1.1)
β_2_Intervention	0.07	0.42	0.871	1.1	(0.47,	2.4)	1.2	(0.56,	2.6)
β_3_Time*Intervention	-0.04	0.05	0.354	0.96	(0.88,	1.1)	0.97	(0.90,	1.1)
Region-level variance	0.07	0.10							
District-level variance	0.54	0.20							
Facility-level variance	0.16	0.30							
**Comparison product 2** (n = 999): Artemisinin-based combination therapy for babies									
β_0_	-1.99	0.70							
β_1_Time	0.02	0.04	0.523	1.0	(0.95,	1.1)	1.0	(0.94,	1.1)
β_2_Intervention	-0.27	0.36	0.460	0.76	(0.37,	1.6)	0.85	(0.40,	1.8)
β_3_Time*Intervention	0.00	0.07	0.995	1.0	(0.88,	1.1)	1.0	(0.92,	1.1)
Region-level variance	0.50	0.41							
District-level variance	0.00	0.00							
Facility-level variance	3.23	1.26							

^a^ Accounting for clustering by facility, district, region (random effects), and zone (fixed effect) and adjusted for standardized sampling weights, which account for effective cluster size [19].

^b^ Contraceptives were combined pill, progesterone-only pill, progesterone-only injectable, implant and intrauterine device.

β: Coefficient; SE: Standard error; OR_stand_: Odds ratio adjusted for standardized sampling weights; OR_raw_: Odds ratio obtained using logistic models accounting for clustering by facility, district, region (random effects), and zone (fixed effect) adjusted for ‘raw’ sampling weights; CI: Confidence Interval for the respective OR; n: number of facility-month observations used in the model; β_0_: Intercept or baseline OR; β_1_Time: Pre-intervention slope or secular trend per month; β_2_Intervention: Change in level after intervention or immediate effect; β_3_Time*Intervention: Post-intervention slope or gradual effect, per month.

### Comparison products

The number of facilities with stock card data for amoxicillin increased from 45 pre-intervention to 59 post-intervention, while there was an increase from 31 to 51 in the number of facilities presenting records for infant ACT (out of 103 facilities). There was no difference in the number of monthly stockout events for our comparison products before and after the implementation of the IPM. Specifically, there were 554 (out of 1,249 facility-month records) stockout events for amoxicillin and 127 (out of 999 facility-month records) for ACT, with 371 (67%) and 87 (69%) of those occurring post-intervention, respectively.

Our analysis confirmed similar odds of stockouts for comparison products pre and post intervention. Confidence intervals for the immediate (β_2_) and gradual (β_3_) effects of IPM all included the null value of one (Table 1). The majority of the variation observed in the odds of ACT monthly stockout (87%) was estimated to be due to characteristics of the health facilities, with remaining variation explained by region characteristics, and none explained by district. In contrast, most variation in the odds of stockout per month for amoxicillin (70%) was attributable to districts characteristics, 21% to facilities’ characteristics, with the rest being explained by region (Table 1).

### Product stockout duration

#### Contraceptive products

**Fig 2:**
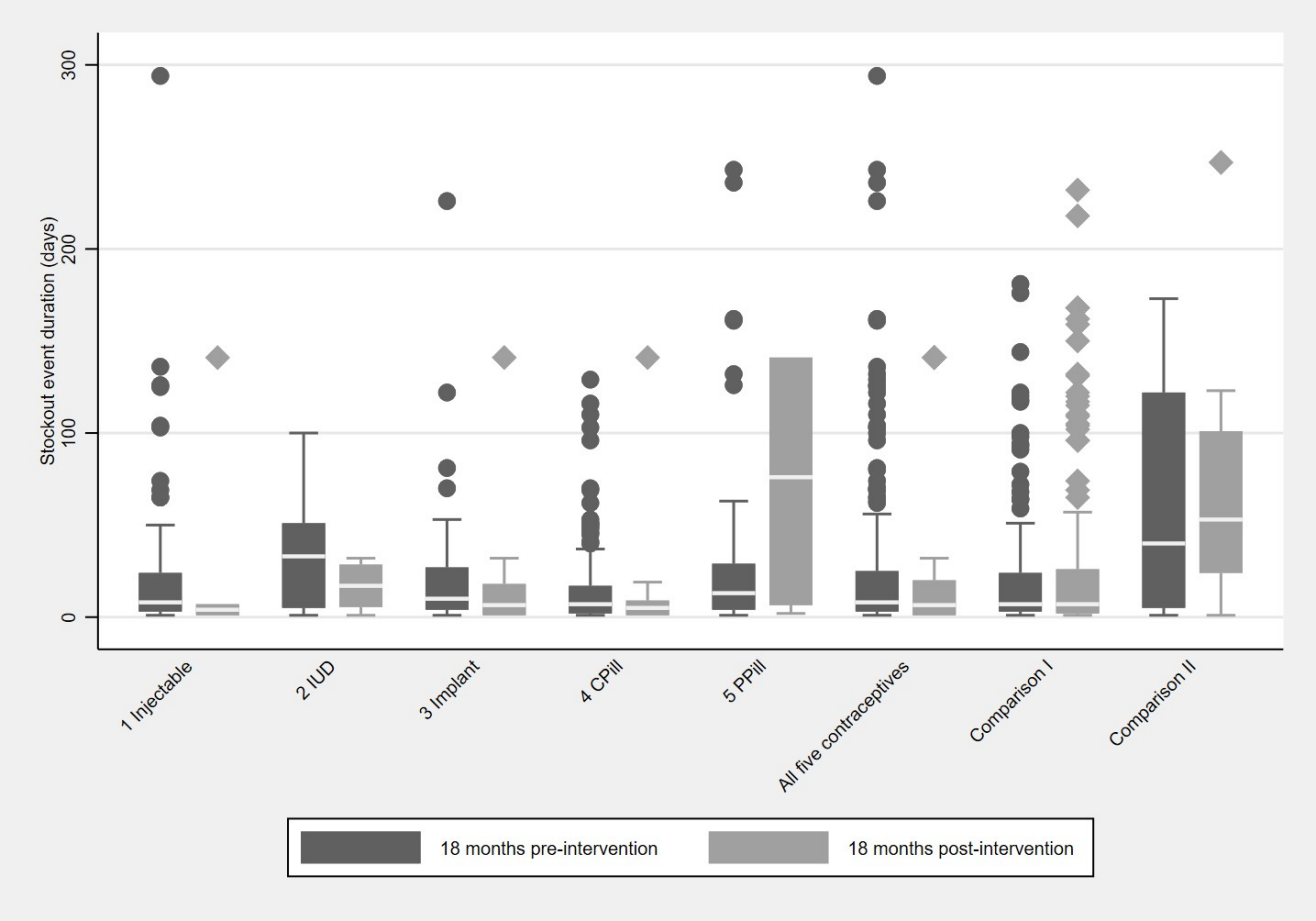
Duration of stockout events per contraceptive product pre- and post-intervention. 103 Senegalese health facilities; Distribution non-adjusted for sampling weights; Contraceptive product (n before, n after): injectable (92, 5), IUD (intrauterine device; 17, 8), Implant (57, 14), CPill (combined pill; 120, 13), PPill (progesterone-only pill; 49, 4), Comparison I (amoxicillin syrup; 121, 216), Comparison II (artemisinin-based combination therapy for babies; 11, 16); Intervention: Informed Push Model; Outliers > 300 d were removed (n = 3 before and n = 4 after the intervention); Very long (> 100 days) stockout durations are probably the result from missing data for several months after a stockout.

During the 18 months before the intervention, there were 335 contraceptive stockout events, with a median duration (interquartile range, **IQR**; not adjusted for sampling weights) of 8 (3–26) days. Following the intervention, there were 44 contraceptive stockout events, with a median (IQR) duration of 6.5 (1–20) days. The distribution of stockout event duration is presented by product, in the form of box-plots (Fig 2). Outlier observations are plotted as individual points. The median duration of stockout events decreased post- intervention compared to pre-intervention for most contraceptives except for progesterone-only pill (Fig 2). Notably, the greatest decrease in stockout duration was for IUD, from a median (IQR) of 41 (5–56) days pre- to 17 (6–29) days post-intervention (see Fig 2). Stockout duration increased for the progesterone-only pill from 13 (4–29) to 76 (7–141) pre- and post-IPM.

There was no evidence of an effect of IPM on stockout duration (IRR, 95%CI: 0.81, 0.24–2.7). It was only when observing the effect of the intervention on duration of stockouts by product (adding an interaction term between IPM and product), that an effect of IPM was observed, and in this case only for long acting contraceptives (Table 2). The model-predicted median (IQR) number of days of stockout for IUD decreased from 22 (14–36) days before, to 4 (1–11) days after the intervention; and from 19 (13–28) to 14 (5–33) days for implants. The variation in stockout duration was only explained by characteristics of the health facilities (Variance proportion = 100%), with none of the variation explained by district or region (Table 2).

**Table 2:** Effect of the Informed Push Model intervention on stockout duration for contraceptive and comparison products in Senegalese health facilities.

Parameter	Level	β^a^	SE^a^	*p*-value^a^	IRR_stand_ (95%CI)^a^			IRR_raw_ (95%CI)		
**The main outcome** (n = 379): Contraceptive products^b^										
**Duration of SO (days)**										
β_0_Intercept		3.16	0.12							
β_1_Intervention		0.52	0.80	0.518	1.7	(0.35,	8.0)	1.5	(0.26,	8.1)
β_2_Product	Injectables	Ref								
	Intrauterine device	1.40	0.34	<0.001	4.1	(2.1,	7.9)	4.2	(2.1,	8.3)
	Implant	0.15	0.20	0.455	1.2	(0.79,	1.7)	1.1	(0.8,	1.5)
	Combined pill	-0.02	0.24	0.938	0.98	(0.61,	1.6)	0.90	(0.55,	1.5)
	Progesterone-only pill	0.49	0.31	0.108	1.6	(0.9,	3.0)	1.7	(1.0,	2.9)
β_3_Intervention*Product	0*Injectables	Ref								
	1*Intrauterine device	-2.64	0.93	0.005	0.07	(0.01,	0.45)	0.08	(0.01,	0.63)
	1*Implant	-0.90	0.41	0.027	0.41	(0.18,	0.91)	0.48	(0.19,	1.2)
	1*Combined pill	-0.75	0.42	0.070	0.47	(0.21,	1.1)	0.55	(0.24,	1.3)
	1*Progesterone-only pill	-0.07	0.57	0.906	0.93	(0.31,	2.9)	1.1	(0.28,	3.9)
Region-level variance		0.00	0.00							
District-level variance		0.00	0.00							
Facility-level variance		3.88	1.15							
**Comparison product 1** (n = 337): Amoxicillin syrup										
β_0_Intercept		3.16	0.09							
β_1_Intervention		0.07	0.18	0.679	1.1	(0.76,	1.5)	1.0	(0.72,	1.5)
Region-level variance		0.00	0.00							
District-level variance		0.00	0.00							
Facility-level variance		0.23	0.13							
**Comparison product 2** (n = 27): Artemisinin-based combination therapy for babies										
β_0_Intercept		4.46	0.45							
β_1_Intervention		0.32	0.43	0.457	1.4	(0.59,	3.2)	1.6	(0.61,	4.0)
Region-level variance		0.02	0.21							
District-level variance		0.00	0.00							
Facility-level variance		0.62	0.26							

^a^ Accounting for clustering by facility, district, region (random effects), and zone (fixed effect) and adjusted for standardized sampling weights, which account for effective cluster size [19].

^b^ Contraceptive products were combined pill, progesterone-only pill, progesterone-only injectable, implant and intrauterine device.

β: Coefficient; SE: Standard error; IRR_stand_: Incidence rate ratio adjusted for standardized sampling weights; IRR_raw_: Incidence rate ratio accounting for clustering by facility, district, region (random effects), and zone (fixed effect) and adjusted for ‘raw’ sampling weights; CI: Confidence Interval for the respective incidence rate ratio; n: number of stockout events used in the model; Ref: reference level; 0: Time pre-intervention; 1: Time post-intervention.

### Comparison products

Stockout durations for the comparison products were very similar pre- and post-intervention (Fig 2). Although ACT stockouts were rare before (n = 11) and after (n = 16) IPM, the median (IQR) duration was 69 (5–149) days pre- and 89 (26–185) days post-intervention. Stockouts were more common for amoxicillin syrup (n = 121 before and n = 216 after IPM), and the median (IQR) duration of a stockout event was 7 (3–24) days before and 6 (2–26) after the IPM.

The regression analysis found no evidence of a difference in stockout duration pre and post-intervention for both amoxicillin (IRR, 95%CI: 1.1, 0.76–1.5) and ACT infant (IRR, 95%CI: 1.4, 0.59–3.2; Table 2). The variation in the duration of stockout events was exclusively explained by characteristics of the health facilities (variance proportion = 100%) for amoxicillin, but with some small proportion being also due to characteristics of region for ACT (variance proportion = 3%).

## Discussion

This is the first nationally representative evaluation of the effect of IPM on contraceptive stockouts in Senegal. The odds of having all methods in stock at the health facilities tripled after the IPM intervention, demonstrating its remarkable success in reducing stockouts of contraceptives. Results from our study confirm findings from other sub-national or shorter term evaluations of the positive effect of IPM on improving the availability of contraceptives in public health facilities [3, 13]. For example, Daff et al. [3] estimated that pre-IPM pilot stockout rates of 14–86% were reduced to 0% post-intervention. While Benson et al. [24] reported in 2011 (pre-IPM) that 68% of women interviewed (6,435 / 9,421) experienced contraceptive stockouts at their closest facility compared to 14% (942 / 6,927) in 2015 after IPM was implemented.

Based on our findings, we hypothesize that one potential contributory factor to the success of IPM for reducing stockouts may have been the removal of the need for health facility personnel to collect supplies from the district level. This could have led to stock standardization throughout districts of the same region. Looking at results for amoxicillin as a proxy for the pre-existing supply chain, results indicate that district level factors accounted for the high level of stockouts of that product, where this was not the case for contraceptives. Meaning that stockouts varied similarly between facilities that were within the same district. For the pre-existing supply chain, district level workers are required to collect supplies from regional warehouses and subsequently, facility level workers collect supplies from district stockrooms. Thus it might be that some districts were better at getting supplies from the higher levels, compared to others, which led to higher or lower stockout events within the health facilities covered by that district. Nevertheless, the IPM intervention included several components in addition to the direct delivery of stock to health facilities. As such it is difficult to point to one factor alone as attributable to its success.

While IPM was successful in reducing stockouts, our analysis found that it was not effective in reducing the duration of contraceptive stockouts, except for long-acting contraceptives. Our model predicted a decrease in stockout median duration from 23 pre- to 4 days post-intervention for IUD; and from 19 to 14 days for implants. This may be because long-acting contraceptives, while they are key products in the MoH drive to increase mCPR, are still not commonly used in Senegal and require trained personnel for their insertion. Their inclusion into IPM meant that facilities were supplied with products that were not in high demand and thus likely to remain on pharmacy shelves.

We could hypothesize that the lack of effect of IPM on stockout duration of short-acting contraception was due to IPM being a preventive rather than a reactive system. Since logisticians would obligatorily pass by facilities once a month after the implementation of the IPM, the stockout duration would only be shorter for those products that had extremely long stockout durations (i.e. greater than one month) prior to the intervention (i.e. IUD). However, when a stockout occurred post-intervention (due to unpredicted spikes in consumption or unplanned outreach activities) [13], workers at the health facility were told that they could call private operators for a top-up. However, our results indicate either a lack of flexibility from private operators in responding to unpredicted stockouts or a lack of communication from health facilities to private operators indicating the need for more stock. Exceptionally, long stockout durations could have been caused by very hard to access health facilities due to seasonal flooding, where stock was delivered to the district to be picked up by the health facility staff. Finally it is also possible that our model did not have the power to detect small changes in the duration of stockouts pre to post-intervention for products that had already lower duration of stockouts.

Variation in stockout events and in duration of stockouts was mainly due to characteristics of health facilities for all products, except for the comparison product amoxicillin. These facility characteristics might include number of personnel available to deal with stock, quality of the stock data produced, and number of personnel qualified to manage contraceptive logistics, among others. Identifying these facility factors could help to improve the IPM supply system.

Use of routine stock card data is rare [15] and was a major strength of our study, providing a comprehensive picture of approximately 36 months of contraceptive turnover across 103 health facilities covering most Senegalese regions. The main reason for lack of use of stock card data prior to IPM, is that facility-level data is paper-based and requires manual compilation and aggregation (if it is compiled at all). However, data availability overall and post-intervention in particular is relatively complete (Fig 1). The absence of digitized data has made it difficult to access and analyse this information by organisations working on supply chains. Nevertheless, routine stock card data also led to some data artefacts such as long (> 100 days) stockout durations (see outliers in Fig 2) resulting from missing data for several months after a stockout. A system to monitor stock reliability and validity, perhaps as part of integrated supervision undertaken by district levels could not only improve the use of stock cards but also the quality of data therein.

We used an interrupted time series analysis to evaluate the impact of IPM on the odds of stockouts. The interrupted time series analysis has become a fundamental study design for the evaluation of public health interventions such as the IPM. The quasi-experimental nature of this type of analysis leads to low possibility of selection bias (i.e. the facilities being compared before and after the intervention are the same) or confounding. One drawback of interrupted time series analysis is the potential for observing an effect due to other concurrent events to the intervention of interest. Of note, is that the IPM intervention was implemented alongside several other strategies aimed at improving mCPR in Senegal. These included health system strengthening programmes that used intense supervision to increase quality of care at facility levels and community level communication and education on the benefits of contraceptives for families [25]. We used comparison products, ACTs and Amoxicillin being distributed through the pre-existing supply chain, to exclude alternative explanations for the effect observed. This increased our study’s validity by analysing the effects of IPM on comparison products (amoxicillin and ACT) not included in the intervention [23], which allow us to account to some degree for other changes (e.g. quality of health facility, seasonality), apart from the intervention, that could have caused observed changes in contraceptive stock [26].

We observed more stockout events for amoxicillin than for ACT, which was expected since antimalarials such as ACT are supplied by the Global Funds [27], and therefore receive more supervision and attention as a commodity compared to amoxicillin. However, amoxicillin is the antibiotic of choice in cases of pneumonia and diarrhoea, and pneumonia is highly incident in Senegal (incidence of 200 clinical cases per 1000 children-year, in children aged under-five years old) [28], accounting for one of the main causes of death within children in sub-Saharan Africa [29].

The IPM did not only ensure stock availability, but also stock data. It is possible, however, that the eligibility criteria for facility selection (S1 File) could have led to some selection bias towards facilities containing more stock data than the average Senegalese health facility. In addition, health facilities with records available are also more likely to better manage their contraceptive stock before the IPM, and therefore the actual impact of IPM on odds of stockouts might be slightly underestimated in our study. Further research and analysis would be required to quantify the correlation between stock data availability and stock availability.

The increase in contraceptive availability as a result of IPM is essential to greater contraceptive access, uptake, and continuation by women in Senegal. A diversity of contraceptive methods increases the chance that users will find the method that best fits their needs. Several studies have shown an association between contraceptive stock availability and use [5, 6]. Within the IPM context, some studies also reported an increase in contraceptive product sales after the intervention [3, 13]. However, it is difficult to say that the increase was exclusively due to the increase in product availability given that IPM was one of several strategies being employed at the time [3, 4].

There are several implications of our analyses. IPM’s success in increasing stock availability for contraceptives led to the subsequent inclusion of additional products into an evolved model referred to as ‘Yeksi Naa’ (“I have arrived”) that delivered products to district level [30]. It is important to note that IPM is a complex intervention and as such some reflection is needed as to the specific component that led to its success. For example, our results suggest that pushing products to facilities could have been a contributory factor. However, what is less clear is how important was it for facilities to have a credit of commodities that was recuperated by district level after products were sold or the intense supervision and oversight of both the private operators and health system components.

## Conclusions

This paper addresses a key gap in the existing literature about the effectiveness of IPM on stock availability and stockout duration. We conclude that the IPM has resulted in greater efficiency in contraceptive stock management, resulting in all five contraceptive products being almost universally available in health facilities at all times since the intervention was implemented, representing a substantial achievement for the MoH and implementation partners. However, the IPM did not appear to reduce the duration of stockouts for contraceptive products, with the exception of long-acting contraception. Duration of stockouts is key to understanding the impact of stock availability on indicators such as Couple Years of Protection and as such, should be included as a key indicator to monitor the effect of supply chain interventions on stock availability.

Given the complexity of the IPM intervention, the factors that led to its success need to be more clearly understood before scaling up to other countries. Further evaluations of supply chain interventions should include comprehensive mapping of intervention components to avoid the risk of implementing a simplified version that does not produce the same results as seen in Senegal. Nevertheless, improving accessibility and availability of multiple contraceptive methods through the IPM should support the government of Senegal to reduce unmet need for contraception and achieve their target of 45% contraceptive prevalence by 2020 [31].

## Supporting information

S1 File (DOCX)

S2 File (DOCX)

S3 File (DOCX)

## Abbreviations

ACT: Artemisinin-based combination therapy
IPM: Informed Push Model
IQR: Interquartile range
IRR: Incidence rate ratio
IUD: Intrauterine device
mCPR: Modern contraceptive prevalence rate
MoH: Ministry of health
OR: Odds ratio
