# Supplementary material for: Evaluation of Senegal supply chain intervention on contraceptive stockouts using routine stock data
Source: PLoS One. 2020 Aug 3;15(8):e0236659. doi: 10.1371/journal.pone.0236659 (PMC7398546; doi:10.1371/journal.pone.0236659)
Supplement: S1 File — (DOCX) [file pone.0236659.s001.docx]

# S1 Data collection

Each team was equipped with one desktop A3 scanner, one uninterruptible power supply battery, an A4 handheld scanner and a back-up digital camera. The team scanned all product stock data available from 24 months pre- to 24 months post-intervention using handheld scanners. In each health facility, the in-charge nurse was first informed about the evaluation work and their consent requested. Once the consent was provided, stock cards were identified and assessed to determine whether enough data was available to include the facility into the sample (i.e. having data for at least 12 months post-intervention and, for service delivery point (**SDP**) stocking contraceptives pre-intervention, having stock cards available at least 12 months pre-intervention). A facility was ultimately defined as having enough data if it had stock cards for at least two of the following five contraceptives: intrauterine device, implant, injectable, combined and progesterone-only contraceptive pills. If stock cards for only one product were available, the facility was included, but an additional facility was surveyed. If insufficient data was available, a replacement SDP was selected at random within the same district. If the replacement facility had stock card availability for only one product or insufficient data, it was excluded, and no further SDP were surveyed.

The products of interest in the current study were not only contraceptive products, but also several potential comparison or tracer products (control or secondary outcome) delivered by the pre-existing supply chain and therefore not exposed to the intervention. There was available information for several potential comparison: Amoxicillin (in syrup and in capsules); Artemisinin-based combination therapy (for babies, older children, teenagers and adults); rapid diagnostic tests for Malaria; iron tablets; and oral rehydration salts. Among these, Amoxicillin in syrup and Artemisinin-based combination therapy for babies were products for which most stock data was available, and were, therefore, selected as comparison outcomes.
