# Supplementary material for: Evaluation of Senegal supply chain intervention on contraceptive stockouts using routine stock data
Source: PLoS One. 2020 Aug 3;15(8):e0236659. doi: 10.1371/journal.pone.0236659 (PMC7398546; doi:10.1371/journal.pone.0236659)
Supplement: S2 File — (DOCX) [file pone.0236659.s002.docx]

# S2 Sampling weights

Since an equal number of regions / districts / facilities were selected from each zone / region / district, independently of the total number of units within each sampling unit, the probability of selection differed between facilities, which has to be accounted for in the analyses (1). To calculate sampling weights, information on Senegal administrative areas was obtained from Senegal Ministry of Health and United States Agency for International Development (2012), on number of regions, districts per regions and service delivery point (i.e. facility) per district. Since hospitals were not considered in the definition of source population, their number was subtracted from the total number of SDP. The probability of selection for any given health facility from each Senegalese zone was calculated as follows:

$$p\left( selection \right)=\frac{r}{R}\times\frac{d}{D}\times\frac{f}{F}$$

where r is the number of regions that were selected from the zone and R is the number of regions in that zone, d is the number of districts that were selected from the sampled region, D is the number of districts in that region, f is the number of facilities that were selected from the sampled districts, and F is the number of facilities in that district. Weighted estimation of multilevel models requires weights at each level of the multilevel data structure (2). Level‐1 weights (facility level): inverses of conditional probabilities of selection, given that a Level‐2 and a Level-3 cluster were sampled (probability calculated as shown in Equation 3). Level‐2 weights (district level): inverses of conditional probabilities of selection, given that a Level-3 cluster was sampled (p = r/R * d/D). Level‐3 weights (regional level): inverses of conditional probabilities of selection (p = r/R).

### References

1. Carle AC. Fitting multilevel models in complex survey data with design weights: Recommendations. BMC Med Res Methodol. 2009; 9:49. <https://doi.org/10.1186/1471-2288-9-49> PMID: 19602263

2. Rabe‐Hesketh S, Skrondal A. Multilevel modelling of complex survey data. J Royal Stat Soc A. 2006; 169:805-27. <https://doi.org/10.1111/j.1467-985X.2006.00426.x>
