# Supplementary material for: Evaluation of Senegal supply chain intervention on contraceptive stockouts using routine stock data
Source: PLoS One. 2020 Aug 3;15(8):e0236659. doi: 10.1371/journal.pone.0236659 (PMC7398546; doi:10.1371/journal.pone.0236659)
Supplement: S3 File — (DOCX) [file pone.0236659.s003.docx]

# S3 Power calculations

## Stockout monthly occurrence

We planned to obtain monthly time series on the odds of stockouts from 18 before to 23 months after the intervention. This would yield n = 41 observations, with the intervention occurring at T = 19, leading to approximately equal pre- and post-intervention time periods. According to Zhang et al. (1), power increases when sample size or effect size increase and it decreases when autocorrelation increases. Assuming a confidence level of 95%, a n = 41, equal pre- and post-intervention time periods and an effect size of 1 (i.e. assuming a 70% probability of guessing the intervention period – before or after – at which a facility was by knowing its odds of stockout) (2) of the effect of IPM on the contraceptive stockouts, the power could vary between 53% and 100%. Lower or higher power would depend on higher or lower autocorrelation, respectively (1). Clustering of observations was not considered for these calculations; therefore, the power presented is likely to be slightly overestimated.

## Stockout duration

Using the STATA statistical software, version 14 (StataCorp), we determined the total number of observations required before and after the IPM intervention. For this analyses, we considered that 95% of the stockout events would last between 1 and 30 days, (4σ = 30 days), which would lead to a standard deviation (σ) of 7.5 days (30 / 4 = 7.5). In addition, we assumed that the total number of stockout events (i.e. sample size) after the IPM would be 15% of the total number before the intervention, based on the results from Daff et al. (3), which showed an impressive decrease after compared to before the IPM. With an alfa of 5% and a power of 80%, we would need 214 stockout events happening before and 33 happening after the intervention if we wanted to detect a difference of at least 4 days in the duration of stockout events before versus after the IPM intervention.

### References

1. Zhang F, Wagner AK, Ross-Degnan D. Simulation-based power calculation for designing interrupted time series analyses of health policy interventions. J Clin Epidemiol. 2011; 64:1252-61. https://doi.org/10.1016/j.jclinepi.2011.02.007 PMID: 21640554

2. Coe R. It's the Effect Size, Stupid - What effect size is and why it is important. Annual Conference of the British Educational Research Association; 12-14 September 2002; University of Exeter, England 2002.

3. Daff BM, Seck C, Belkhayat H, Sutton P. Informed push distribution of contraceptives in Senegal reduces stockouts and improves quality of family planning services. Glob Health Sci Pract. 2014;2:245-52. <https://doi.org/10.9745/GHSP-D-13-00171> PMID: 25276582
